# O Papel do Escore Prognóstico de Nápoles na Avaliação da Permeabilidade do EVS após Cirurgia de Revascularização do Miocárdio

**DOI:** 10.36660/abc.20250182

**Published:** 2025-05-29

**Authors:** Mustafa Ozan Gürsoy, Hakan Güneş

**Affiliations:** 1 İzmir İl Sağlık Müdürlüğü İzmir Sağlık Bilimleri Üniversitesi Tepecik Eğitim ve Araştırma Hastanesi Konak Turquia İzmir İl Sağlık Müdürlüğü İzmir Sağlık Bilimleri Üniversitesi Tepecik Eğitim ve Araştırma Hastanesi, Konak – Turquia; 2 Ministry of Health Sciences University Izmir Tepecik Education and Research Hospital Cardiology Konak Turquia Ministry of Health Sciences University Izmir Tepecik Education and Research Hospital – Cardiology, Konak, İzmir – Turquia

**Keywords:** Prognóstico, Veia Safena, Permeabilidade

As doenças cardiovasculares são responsáveis por aproximadamente um terço das mortes no mundo, e a doença cardíaca isquêmica é a forma mais comum.^1^ A cirurgia de revascularização do miocárdio (CRM) é um procedimento comum realizado no tratamento da doença arterial coronariana, mas as menores taxas de permeabilidade dos enxertos são as principais limitações durante o acompanhamento de curto e longo prazo.^2^ A falha do enxerto varia de 10% a 50%, dependendo do tipo de conduto utilizado, com maior incidência encontrada em enxertos de veia safena (EVS).^3^

Mediadores inflamatórios desempenham papéis essenciais no desenvolvimento da aterosclerose^4^ e têm sido extensivamente estudados em vários distúrbios cardíacos e não cardíacos.^1 , 5^ O escore prognóstico de Nápoles (NPS) é um novo escore desenvolvido de acordo com o estado inflamatório e nutricional que tem sido frequentemente estudado em oncologia, especialmente em cânceres gastrointestinais.^5^ Também foi proposto como um biomarcador inflamatório e potencial preditor de risco e prognóstico em pacientes com doença arterial coronariana, incluindo infarto do miocárdio com supradesnivelamento do segmento ST (IAMCSST).^1^ O NPS pode fornecer informações sobre a condição inflamatória e nutricional de um paciente, incluindo a razão linfócito-monócito (RLM), razão neutrófilo-linfócito (RNL), colesterol total (CT) e níveis séricos de alfa. O CT e a albumina sérica refletem o estado nutricional do corpo, enquanto a RNL e a RLM representam sua condição imunoinflamatória. Isso permite uma avaliação mais completa do estado físico do paciente.^6^

A falha dos EVSs após CRM é uma preocupação significativa devido à sua associação com eventos adversos e preditores, e esforços anteriores foram feitos para identificar preditores potenciais para a permeabilidade do enxerto usando vários biomarcadores, incluindo níveis de largura de distribuição de plaquetas, proporção de plaquetas e linfócitos, RNL e níveis de ácido úrico.^7 - 11^ Na edição atual do ABC Cardiol, Karaduman et al.^12^ investigou a relação potencial entre NPS e doença EVS em pacientes com histórico de cirurgia de revascularização do miocárdio. Como o NPS era uma combinação de vários parâmetros inflamatórios e nutricionais, ele foi considerado como tendo um importante papel preditivo em comparação a outros biomarcadores individuais. Os autores demonstraram que o NPS foi maior no grupo com degeneração da veia safena e emergiu como um preditor significativo para doença do EVS, juntamente com diferentes parâmetros, incluindo hipertensão, doença renal crônica, intervalo de tempo desde a cirurgia de revascularização do miocárdio e número de EVSs. Neste estudo, a população de pacientes foi heterogênea: um terço dos pacientes apresentava doença arterial coronariana estável, enquanto o restante havia sofrido síndrome coronariana aguda (principalmente Não-IAMCSST). Isso pode ter influenciado parcialmente a interpretação dos resultados, mas já foi mencionado como uma limitação.

Resultados conflitantes também estão presentes na literatura atual quanto ao papel do NPS na predição da doença arterial coronariana. Recentemente, Ozkan et al.^13^ estudaram 1.138 pacientes submetidos à angiotomografia coronariana e descobriram que o índice de imunoinflamação sistêmica (IIS) pode predizer a gravidade da doença arterial coronariana, enquanto o NPS pode não ter esse efeito. Eles sugeriram que a presença de outros parâmetros que afetam os níveis de albumina e colesterol total pode ter impedido uma correlação significativa com a síndrome coronariana crônica. Deve-se ter em mente que o estado nutricional, a função hepática e o equilíbrio de fluidos corporais podem ter um efeito considerável nos níveis de albumina.^14^ Além disso, os níveis de colesterol total também podem ser afetados por hábitos alimentares de curto prazo. Ozkan et al. também sugeriram que não usar o valor do LDL-C pode ter reduzido o poder preditivo do NPS,^13^ já que o LDL é considerado o principal fator de risco para aterosclerose; essas descobertas mostram que, embora o NPS tenha um papel prognóstico incremental em muitos distúrbios oncológicos e cardíacos, ele ainda tem algumas limitações e pode necessitar de algumas atualizações futuras.

Consequentemente, o NPS, calculado a partir dos valores de RNL, RLM, albumina e CT, demonstrou ser mais sensível do que esses marcadores individualmente. Com base na publicação atual,^12^ o NPS pode oferecer novos insights sobre a permeabilidade do EVS após cirurgia de revascularização miocárdica. Por outro lado, a precisão do escore pode ser influenciada por fatores de confusão, como a presença de síndrome coronariana aguda ou outros fatores que podem afetar as respostas inflamatórias e metabólicas. Portanto, estudos futuros em larga escala sobre doenças cardiovasculares são necessários para confirmar o valor do NPS como um biomarcador inflamatório e nutricional.

## References

[1] Birdal O, Pay L, Aksakal E, Yumurtaş AÇ, Çinier G, Yücel E (2024). Naples Prognostic Score and Prediction of Left Ventricular Ejection Fraction in STEMI Patients. Angiology.

[2] Harik L, Perezgrovas-Olaria R, Soletti G, Dimagli A, Alzghari T, An KR (2023). Graft Thrombosis after Coronary Artery Bypass Surgery and Current Practice for Prevention. Front Cardiovasc Med.

[3] Buxton BF, Hayward PA, Raman J, Moten SC, Rosalion A, Gordon I (2020). Long-Term Results of the RAPCO Trials. Circulation.

[4] Libby P, Okamoto Y, Rocha VZ, Folco E (2010). Inflammation in Atherosclerosis: Transition from Theory to Practice. Circ J.

[5] Nakagawa N, Yamada S, Sonohara F, Takami H, Hayashi M, Kanda M (2020). Clinical Implications of Naples Prognostic Score in Patients with Resected Pancreatic Cancer. Ann Surg Oncol.

[6] Unkun T, Fidan S, Derebey ST, Şengör BG, Aytürk M, Sarı M (2024). The Value of Naples Prognostic Score in Predicting Ischemia on Myocardial Perfusion Scintigraphy. Koşuyolu Heart J.

[7] Tavil Y, Sen N, Hizal F, Açikgöz SK, Taşoğlu I, Topal S (2008). Relationship between Elevated Levels of Serum Uric Acid and Saphenous Vein Graft Disease. Turk Kardiyol Dern Ars.

[8] Ege MR, Guray U, Guray Y, Acıkgoz S, Demirkan B (2013). Platelet Distribution Width and Saphenous Vein Disease in Patients after CABG. Association with Graft Occlusion. Herz.

[9] Akpinar I, Sayin MR, Gursoy YC, Karabag T, Kucuk E, Buyukuysal MC (2014). Plateletcrit. A platelet Marker Associated with Saphenous Vein Graft Disease. Herz.

[10] Yayla Ç, Canpolat U, Akyel A, Yayla KG, Yilmaz S, Açikgöz SK (2016). Association between Platelet to Lymphocyte Ratio and Saphenous Vein Graft Disease. Angiology.

[11] Oksuz F, Elcik D, Yarlioglues M, Duran M, Ozturk S, Celik IE (2017). The Relationship between Lymphocyte-to-Monocyte Ratio and Saphenous Vein Graft Patency in Patients with Coronary Artery Bypass Graft. Biomark Med.

[12] Karaduman A, Yılmaz C, Tiryaki MM, Balaban I, Keten MF, Unkun T (2025). Relationship Between the Naples Prognostic Score and Saphenous Vein Graft Disease after Coronary Artery Bypass Grafting Surgery. Arq Bras Cardiol.

[13] Ozkan E, Erdogan A, Karagoz A, Tanboğa IH (2024). Comparison of Systemic Immune-Inflammation Index and Naples Prognostic Score for Prediction Coronary Artery Severity Patients Undergoing Coronary Computed Tomographic Angiography. Angiology.

[14] Franch-Arcas G (2001). The Meaning of Hypoalbuminaemia in Clinical Practice. Clin Nutr.

