# Supplementary material for: O Papel do Escore Prognóstico de Nápoles na Avaliação da Permeabilidade do EVS após Cirurgia de Revascularização do Miocárdio
Source: Arq Bras Cardiol. 2025 May 29;122(5):e20250182. [Article in Portuguese] doi: 10.36660/abc.20250182 (PMC12212393; doi:10.36660/abc.20250182)
Supplement: Translation

# Translation: The Role of the Naples Prognostic Score in the Evaluation of SVG Patency Following CABG Surgery

**DOI:** 10.36660/abc.20250182i

**Authors:** Mustafa Ozan Gürsoy, Hakan Güneş

**Affiliations:** 1 İzmir İl Sağlık Müdürlüğü İzmir Sağlık Bilimleri Üniversitesi Tepecik Eğitim ve Araştırma Hastanesi Konak Türkiye İzmir İl Sağlık Müdürlüğü İzmir Sağlık Bilimleri Üniversitesi Tepecik Eğitim ve Araştırma Hastanesi, Konak – Türkiye; 2 Ministry of Health Sciences University Izmir Tepecik Education and Research Hospital Cardiology Konak Türkiye Ministry of Health Sciences University Izmir Tepecik Education and Research Hospital – Cardiology, Konak, İzmir – Türkiye

**Keywords:** Prognosis, Saphenous Vein, Permeability

Cardiovascular diseases are responsible for approximately one-third of deaths worldwide, and ischemic heart disease is the most common form.^1^Coronary artery bypass grafting (CABG) is a common procedure performed in the management of coronary artery disease, but the lower patency rates of grafts are the major limitations during the short and long-term follow-up.^2^The graft failure ranges from 10%–50%, depending upon the type of conduit used, with the highest incidence found in saphenous vein grafts (SVGs).^3^

Inflammatory mediators play essential roles in the development of atherosclerosis^4^and have been extensively studied in various cardiac and noncardiac disorders.^1 , 5^The Naples Prognostic Score (NPS) is a novel score developed according to the inflammatory and nutritional status that has been frequently studied in oncology, especially gastrointestinal cancers.^5^It has also been proposed as an inflammatory biomarker and potential predictor of risk and prognosis in patients with coronary artery disease, including ST-elevation myocardial infarction (STEMI).^1^NPS may provide information about a patient's inflammatory and nutritional condition, including lymphocyte-to-monocyte ratio (LMR), neutrophil-to-lymphocyte ratio (NLR), total cholesterol (TC), and serum alpha levels. TC and serum albumin reflect the body's nutritional status, whereas NLR and LMR represent its immunoinflammatory condition. This permits a more thorough assessment of the patient's physical state.^6^

The failure of SVGs following CABG is a significant concern due to its association with adverse events and predictors, and previous efforts have been made to identify potential predictors for graft patency using multiple biomarkers, including platelet distribution width levels, platelet lymphocyte ratio, NLR, and uric acid levels.^7 - 11^In the current issue of the ABC Cardiol, Karaduman et al.^12^investigated the potential relationship between NPS and SVG disease in patients with a history of CABG surgery. As NPS was a combination of several inflammatory and nutritional parameters, it was considered to have a major predictive role compared to other individual biomarkers. The authors showed that NPS was higher in the group with saphenous vein degeneration and emerged as a significant predictor for SVG disease along with different parameters, including hypertension, chronic kidney disease, time interval since CABG, and number of SVGs. In this study, the patient population was heterogeneous: One-third of the patients had stable coronary artery disease, whereas the remaining had suffered acute coronary syndrome (mostly Non-STEMI). This might have partially influenced the interpretation of the findings but has already been mentioned as a limitation.

Conflicting results are also present in the current literature regarding the role of NPS in predicting coronary artery disease. Recently, Ozkan et al.^13^studied 1138 patients having coronary computed tomographic angiography and discovered that systemic immune-inflammation index (SII) might predict coronary artery severity, whereas NPS might not. They have suggested that the presence of other parameters that affect albumin and total cholesterol levels may have prevented a significant correlation with chronic coronary syndrome. It should be kept in mind that nutritional status, liver function, and body fluid balance may have a considerable effect on albumin level.^14^Furthermore, TC levels may also be affected by short-term eating habits. Ozkan et al. also suggested that not using the LDL-C value might have reduced the predictive power of the NPS,^13^as LDL is considered the main risk factor for atherosclerosis; these findings show that although NPS has an incremental prognostic role in many oncologic and cardiac disorders, it has still some limitations and may necessitate some future updates.

Consequently, NPS, which is calculated from NLR, LMR, albumin, and TC values, has been demonstrated to be more sensitive than these markers individually. On the basis of the current publication,^12^NPS may offer novel insights into SVG patency after CABG surgery. On the other hand, the accuracy of the score might be influenced by confounding factors such as the presence of acute coronary syndrome or other factors that may affect inflammatory and metabolic responses. Therefore, future large-scale studies on cardiovascular disorders are required to confirm the value of NPS as an inflammatory and nutritional biomarker.
